# Dynamic High-Cadence Cycling Improves Motor Symptoms in Parkinson’s Disease

**DOI:** 10.3389/fneur.2015.00194

**Published:** 2015-09-02

**Authors:** Angela L. Ridgel, Robert S. Phillips, Benjamin L. Walter, Fred M. Discenzo, Kenneth A. Loparo

**Affiliations:** ^1^Department of Exercise Physiology, Kent State University, Kent, OH, USA; ^2^Movement Disorders Center, University Hospital Cleveland, Cleveland, OH, USA; ^3^Department of Neurology, Case Western Reserve University, Cleveland, OH, USA; ^4^Department of Industrial Automation, Rockwell Automation, Mayfield Heights, OH, USA; ^5^Department of Electrical Engineering and Computer Science, Case Western Reserve University, Cleveland, OH, USA

**Keywords:** movement disorders, exercise, rehabilitation, neuroplasticity, bradykinesia, motor function

## Abstract

**Rationale:**

Individuals with Parkinson’s disease (PD) often have deficits in kinesthesia. There is a need for rehabilitation interventions that improve these kinesthetic deficits. Forced (tandem) cycling at a high cadence improves motor function. However, tandem cycling is difficult to implement in a rehabilitation setting.

**Objective:**

To construct an instrumented, motored cycle and to examine if high cadence dynamic cycling promotes improvements in motor function.

**Method:**

This motored cycle had two different modes: dynamic and static cycling. In dynamic mode, the motor maintained 75–85 rpm. In static mode, the rider determined the pedaling cadence. UPDRS Motor III and Timed Up and Go (TUG) were used to assess changes in motor function after three cycling sessions.

**Results:**

Individuals in the static group showed a lower cadence but a higher power, torque and heart rate than the dynamic group. UPDRS score showed a significant 13.9% improvement in the dynamic group and only a 0.9% improvement in the static group. There was also a 16.5% improvement in TUG time in the dynamic group but only an 8% improvement in the static group.

**Conclusion:**

These findings show that dynamic cycling can improve PD motor function and that activation of proprioceptors with a high cadence but variable pattern may be important for motor improvements in PD.

## Introduction

Approximately 630,000 people in the US were diagnosed with Parkinson’s disease (PD) in 2010 and it is estimated that PD prevalence will double by 2040 (1). As PD progresses, the combined motor and non-motor symptoms often lead to decreased independence and quality of life. The economic impact of PD, including treatment, social security payments, and lost income from inability to work, exceeded $14.4 billion in 2010 (1). The degenerative nature of PD results in progressive deterioration of motor skills along with reduced sensory and cognitive function. The current treatment for PD is medication (levodopa, dopamine agonists) and surgical intervention (deep brain stimulation). These treatments only partially treat the symptoms and do not slow progression of the disease. Furthermore, they often have undesirable side effects, such as dyskinesia (2). In light of projections of increased prevalence of PD, there is a need for innovative new treatments to improve symptoms and delay disease progression.

Our studies, and those of several others, have presented strong evidence that certain exercise interventions promote changes in neural drive in PD (3–9). Although the exact mechanisms are still unknown, it has been suggested that increases in sensory input or feedback resulting from these interventions may play a role in this motor improvement. Individuals with PD often have deficits in kinesthesia (conscious awareness of limb and body position in space) (10, 11). Sensorimotor integration may be dysfunctional in PD and has been implicated in the etiology for bradykinesia and atypical movement in PD. Kinesthesia is likely to be the key modality affecting this dysfunction (12). However, levodopa does not appear to improve kinesthetic deficits in PD (13, 14) and has been associated with suppression of sensitivity to joint position (15). Therefore, there is a great need for rehabilitation interventions that improve proprioceptive deficits in PD.

Animal model studies have shown that high-intensity exercise can promote neural plasticity and neuroprotection against dopaminergic cell loss (16). Several reports in humans have shown that high-intensity treadmill training (17, 18) and high-cadence cycling (3, 6, 9, 19, 20) promote functional improvement in PD but there are still several unanswered questions: (1) How does motor function change immediately after high-intensity exercise, (2) What features of exercise (speed, intensity) optimize motor function, and (3) What are potential mechanisms of function improvements after high-intensity exercise?

To begin to address these questions, we have developed a novel rehabilitation approach called dynamic cycling. This work builds upon our original “forced exercise” paradigm that used a stationary tandem bicycle and an able-bodied trainer to assist individuals to pedal with a rapid cadence (80 rpm) (6). High-cadence tandem cycling resulted in a 35% reduction in PD motor symptoms (UPDRS scores), whereas individuals who cycled at a self-selected cadence (60 rpm) showed no improvement. Despite these remarkable results, large-scale use of the tandem cycling paradigm is not feasible in a rehabilitation or home setting. Furthermore, it has proven difficult to reproduce the dynamics of tandem cycling using currently available motorized cycles. The dynamic cycling paradigm that we developed uses a motorized stationary cycle to assist individuals with PD to pedal at a cadence faster than they can (or would) pedal on their own. In addition, this rehabilitation paradigm is unique because the motor rotates the pedals at a high speed with a slight, but prescribed, variation. These dynamic changes in cadence appear to be an important component of tandem cycling (21). Therefore, we hypothesize that dynamic cycling will promote greater improvements in PD symptoms and motor function than cycling at a lower cadence (static cycling). Findings from this study will provide important data to support future research examining long-term rehabilitation benefits as well as role of afferent input during dynamic cycling in the reduction of PD motor symptoms.

## Materials and Methods

### Participants

Inclusion criteria were as follows: 50–79 years of age, diagnosis of idiopathic PD, and no contraindications to exercise, including uncontrolled cardiovascular disease or stroke. Exclusion criteria included history of heart attack, any surgical procedure for treatment of PD, including deep brain stimulation, pallidotomy, or thalamotomy. All potential study subjects were pre-screened over the telephone with the American Heart Association/American College of Sports Medicine exercise pre-participation questionnaire (22). Individuals with greater than or equal to two risk factors for coronary artery disease (moderate risk) were required to obtain physician clearance prior to exercise. Fifty individuals with idiopathic PD qualified and agreed to participate in this study. This study was carried out in accordance with the recommendations of the Kent State University Institutional Review Board with written informed consent from all subjects.

### Study design

This study was a randomized two group pretest–posttest design. Each participant visited the lab for four sessions. Individuals were randomized into either: (1) dynamic cycling or (2) static cycling. During the first session (Friday), baseline motor function was assessed and individuals completed the first cycling session. During the next two sessions (Monday/Wednesday), each participant exercised for 40 min on the instrumented bike. During the last session (Friday), post-intervention motor function was assessed. There was at least 48 h between the last exercise session and the post-intervention assessments. Each cycling bout began with 5 min of warm up (low resistance pedaling at 40–50 rpm). Participants then completed 30 min of dynamic or static cycling and ended with 5 min of cycling at 40–50 rpm. Rating of perceived exertion (RPE) and heart rate (HR) was monitored by a research assistant during each session. Participants were encouraged to maintain their HR within 50–80% of their estimated HR reserve. Data from the cycle were collected continuously during each session. All exercise sessions were completed while individuals were “on” anti-Parkinson’s medications. Participants served as their own controls from pre-cycling to post-cycling testing to account for performance variability that is often present in PD.

### Intervention

During dynamic cycling, motor output speed varied between 75 and 85 rpm. Motor torque was adjusted to accommodate changes in the rider force exerted on the pedals. The motor did the majority of the work to turn the pedals but individuals were encouraged to push on the pedals and to not be passive. During static cycling, individuals cycled on the instrumented bike, at a self-selected speed, without the motor assist. Speed was not controlled but the rider experienced an inertia load on the pedals, similar to what they would experience on a typical stationary bike. Individuals were directed to choose their own pedaling speed. HR was collected with a Polar Wearlink+™ Coded Transmitter worn on the chest, which transmitted to a HR monitor interface board. The control platform was a commercially available programmable logic controller (PLC). The PLC determined the appropriate motor speed and load (torque) values, and sent motor control information to the motor drive. The motor drive implemented a high-speed inner loop controller that provided the appropriate voltage and current to the motor. Motor feedback was used as feedback for the drive to maintain motor speed and torque. Data from the controller box were downloaded and archived onto a laptop computer. Additional details on the design of the control program and the cycle can be found in the paper by Mohammadi Abdar (23).

### Outcome measures

All assessments were completed while the individuals were “on” anti-Parkinson’s medication. The primary outcome measure was the UPDRS Part III Motor Exam. UPDRS Motor III was administered by a blinded movement disorders specialist prior (pre-intervention) to the three cycling sessions and 2 days following the last exercise session (post-intervention). The UPDRS Motor III has universal acceptance as a rating scale for PD patients and it has been shown to be reliable and valid (24, 25). The total score, scores for each primary symptom (i.e., tremor, bradykinesia) and scores for upper and lower extremity were analyzed. The secondary outcome measure was the Timed Up and Go (TUG). This test is used primarily as a measure of mobility but is also useful as a measure of bradykinesia during walking (26). To complete the TUG, participants were asked to stand up from a standard chair and walk a distance of 3 m, turn around and walk back to the chair and sit down again. The time to complete the task was recorded with a stop watch. Each participant performed three trials and the average was calculated.

### Statistics

Demographic variables between the two groups were compared using an independent samples *t*-test. Comparison of pre-cycling and post-cycling changes in UPDRS motor scores and TUG time were performed using paired-samples *t*-test in each group (dynamic, static) independently. All statistical analysis was completed using SPSS V. 22 and the alpha level was set to 0.05.

## Results

**Figure 1:**
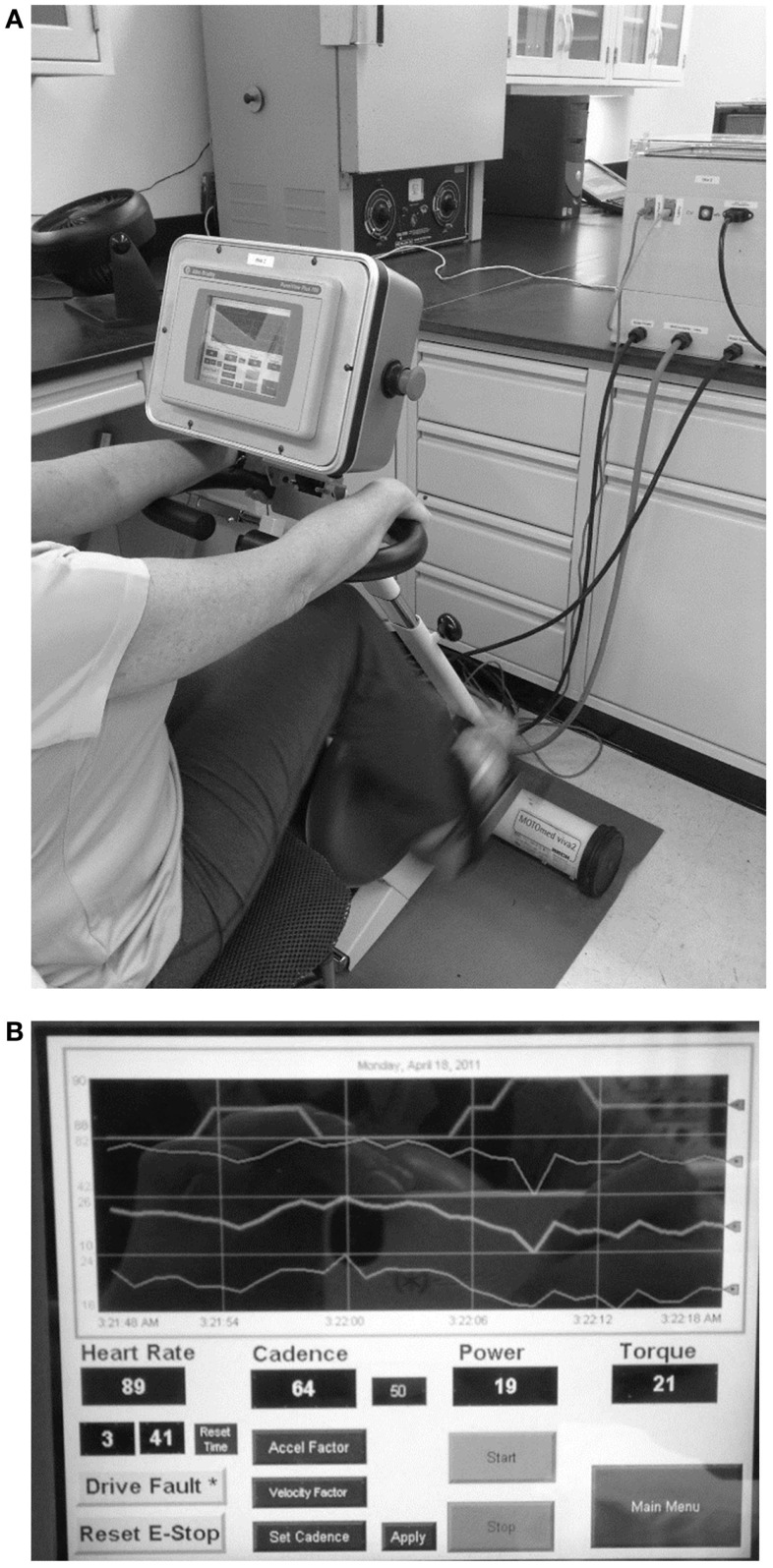
**Dynamic/static motorized cycle design (A) A motorized stationary movement trainer (Motomed Viva 2, Reck, LLC) provided the mechanical chassis for the bike**. This device was modified by replacing the motor and by adding a controller box. Details of the design of the controller are described in the text. **(B)** A touch screen (PanelView™ Plus graphic display) was also added to allow for visual feedback for the subjects and the research assistants.

A detailed description of the design and controller parameters for the motorized cycle was described in a previous paper (23). In summary, the bike chassis used for this study consisted of a commercial exercise bike frame (Motomed Viva 2, Reck, Germany) that was modified to include additional sensors to monitor bike operation and rider condition (cadence, torque, power, HR). In addition, a high performance servomotor and variable speed drive were coupled to the pedals and a programmable controller with custom control algorithms, data acquisition, network capability, real-time display with operator controls, and data archiving were provided (Figure 1A). The electronic components integrated on the bike chassis were the operator display, emergency stop button, HR monitor interface board, TTL to serial level converter board for the HR monitor. All other electronic components, such as the drive, programmable controller, network adapter, and power supplies, were mounted in an enclosure that was external to the bike but connected via cables for motor power, display power, motor feedback, and communications (Figure 1B).

**Figure 2:**
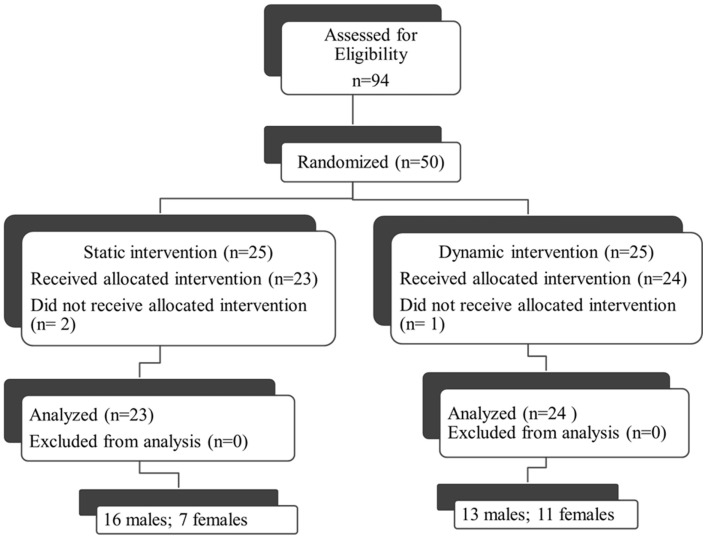
**Consort diagram**. Fifty individuals who qualified for the study (out of 94 assessed, 53%) were randomized to either the dynamic or static cycling group. Two individuals from the static group did not complete the intervention due to DBS (not reported in prescreening) and a diagnosis of a non-Parkinson’s movement disorder (PSP). One person from the dynamic did not complete the protocol due to hip pain during the cycling. Data from 23 individuals in the static group and 24 individuals in the dynamic group were analyzed.

Fifty individuals were randomized to either the dynamic or static cycling group (Figure 2). Seven females and 16 males with mean age 67.3 ± 0.9 years completed three 40-min static cycling sessions and 11 females and 13 males with mean age 67.2 ± 1.6 years completed three dynamic cycling sessions (Figure 2). Two individuals from the static group and one person from the dynamic did not complete the intervention due to the reasons outlined in Figure 2. There were no significant differences in any of the demographic variables (age, H&Y, height, weight, body mass index, disease duration, and levodopa equivalent dose) between the dynamic and static cycling groups (Table 1).

**Table 1:** **Demographic variables**.

Variable	Static (*n* ***=*** 23)	Dynamic (*n* ***=*** 24)	*p*-Value
Ages (years)	67.3 ± 0.9	67.2 ± 1.6	0.962
Male/female	16/7	13/11	–
H&Y (Hoehn and Yahr)	1.8 ± 0.1	2.1 ± 0.2	0.151
Height (cm)	67.7 ± 0.7	68.1 ± 0.8	0.681
Weight (lbs)	165.2 ± 6.0	175.1 ± 8.1	0.336
BMI	25.1 ± 0.7	26.6 ± 0.9	0.186
PD duration (months)	77.7 ± 9.7	83.5 ± 11.2	0.702
Levodopa equivalent dose	153.3 ± 23.9	178.8 ± 29.4	0.507

*Mean ± SD, independent *t*-test was used to compare the two groups*.

Dynamic and static cycling modes resulted in similar individual assessments of RPE but there were significant differences in cadence, power, torque, and HR between the two groups (Table 2). Specifically, individuals in the dynamic cycling group showed a higher cadence (78.6 ± 1.1 versus 66.0 ± 3.2 rpm, *p* < 0.001) but a lower power, torque, and HR than the static cycling group.

**Table 2:** **Cycling and physiological variables**.

Variable	Static (*n* ***=*** 23)	Dynamic (*n* ***=*** 24)	*p*-Value
Cadence (rpm)	66.0 ± 3.2	78.6 ± 1.1	0.000
Power	31.2 ± 4.1	8.0 ± 4.3	0.000
Torque	29.2 ± 2.9	0.2 ± 3.9	0.000
Heart rate (bpm)	103.3 ± 3.1	91.1 ± 2.5	0.004
RPE (6–20 scale)	13.6 ± 0.4	12.7 ± 1.1	0.417

*rpm, revolutions per minute; bpm, beats per minute; RPE, rating of perceived exertion*.

*Mean ± SD, independent *t*-test was used to compare the two groups*.

**Figure 3:**
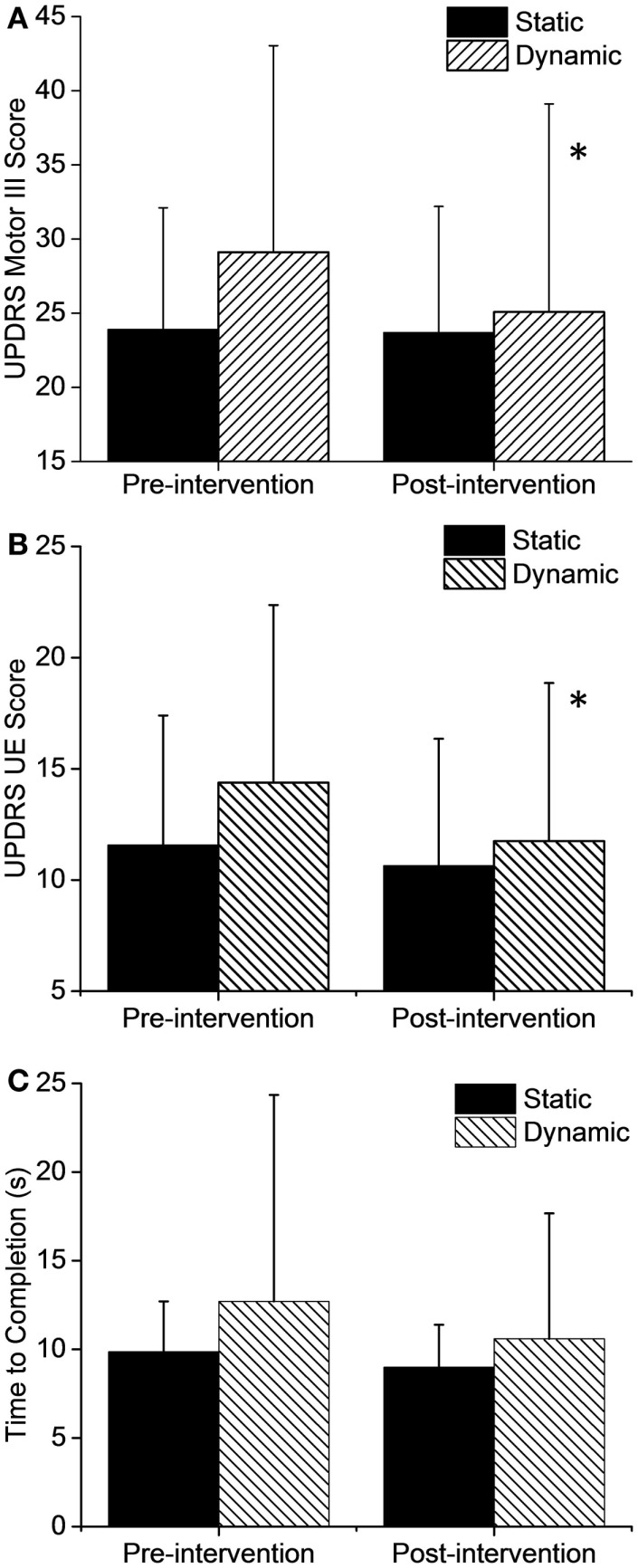
**Changes in motor function after the intervention**. **(A)** Total UPDRS Motor III scores and upper extremity (UE) only **(B)** showed significant improvements after dynamic cycling. **(C)** TUG time to completion did not show a significant change but improvements were greater in the dynamic group by two-fold. Error bars represent SD. *p < 0.05.

The overall UPDRS III score (Figure 3A) showed a significant 13.9% (4.0 pts) improvement in the dynamic group (*t* = 2.676, df = 23, *p* = 0.013) and only a small 0.9% (0.2 pts) change in the static group (*t* = 0.189, df = 22, *p* = 0.85) after just three cycling sessions. Analysis of the individual UPDRS III components showed that lower extremity (*t* = 3.8, df = 23, *p* = 0.001) and rigidity scores (*t* = 2.6, df = 23, *p* = 0.013) also improved significantly in the dynamic group but there were no significant changes in the static group in any of the UPDRS Motor III scores. Interestingly, UPDRS scores in the upper extremity (Figure 3B) showed a significant 18% (2.6 pts) improvement after dynamic cycling (*t* = 2.54, df = 23, *p* = 0.018) compared with a 7% (0.9 pts) improvement in the static group (*t* = 1.32, df = 22, *p* = 0.19).

In addition, there was a 16.5% improvement (2.1 s, *t* = 1.7, df = 23, *p* = 0.10) in Timed Up and Go test (TUG) time in the dynamic group but only an 8% improvement (0.87 s, *t* = 1.3, df = 21, *p* = 0.19) in the static group (Figure 3C). Although this change was not statistically significant due to variability in responses among individuals, it is interesting that the dynamic group showed a twofold improvement in the TUG compared to the static group.

**Figure 4:**
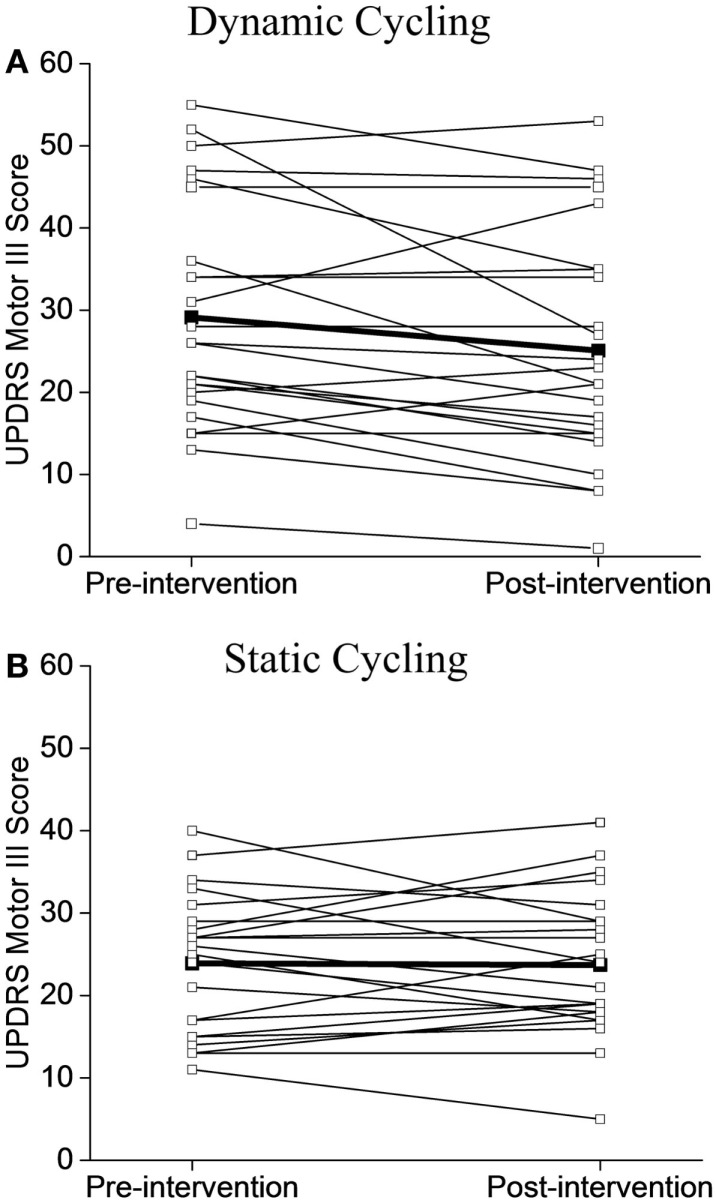
**Individual UPDRS Motor III Scores before and after the intervention**. **(A)** Dynamic cycling group. **(B)** Static cycling group. Individual subject values are illustrated with open circles, group mean values are noted as black squares.

Although the baseline mean scores of UPDRS between the two groups were not the same, the majority of individuals in the dynamic group (15/24, 62%, Figure 4A) showed improvements in motor symptoms and a much smaller percentage of the static group showed a positive change (9/23, 39%, Figure 4B).

## Discussion

Dynamic high-cadence cycling for only three sessions resulted in a 4-point reduction in motor symptoms of PD as measured with the UPDRS Motor III test. These findings were similar to a 1-month high-intensity treadmill training program (body weight supported treadmill training, BWSTT) which resulted in a 3-point improvement in UPDRS Motor III (18) and an 8-week high-intensity BWSTT intervention which showed a 2.8-point improvement. In addition, an intensive 4-week LSVT ^®^BIG intervention reported a 5-point change in UPDRS Motor III (27). By contrast, our UPDRS improvements were less than that reported after a single session of forced cycling (as evaluated “off” medication) (3). However, a 4-point change is well within the minimum clinically important difference (CID) of 2.3–2.7 points (28), suggesting that this difference is recognized and valuable to individuals with PD. UPDRS Motor III scores were evaluated while individuals were “on” medication in this study (see Discussion below), so we expect that future studies with a longer intervention and with “off” medication evaluations would yield even greater improvements. The significant changes seen in upper extremity UPDRS scores after dynamic cycling (2.6 points) are in agreement with previous high-cadence cycling papers (3, 6). The enhanced function of the upper extremity with a lower extremity intervention further supports the view that dynamic cycling could promotes changes in neural drive in PD.

Timed up and go is a widely used measure of mobility, balance, and fall risk in PD. Time to completion in the TUG test improved by 2.1 s after dynamic cycling but variability among subjects resulted in a non-significant difference. The minimal detectable change (MDC), difference in scores which reflect true change and not error, for PD is reported as 3.5 s (29). However, Latt and colleagues (30) reported that individuals who complete the TUG in ≥12 s have an increased fall risk. Individuals in the dynamic group had a baseline TUG of 12.7 ± 11.6 s that was decreased to 10.5 ± 7.1 s after only three dynamic cycling sessions. Furthermore, it is likely that a longer-term intervention would promote greater improvements in balance and mobility.

### Dynamic cycling versus high-intensity exercise

Dynamic cycling promoted similar improvements in motor function to that reported in other high-intensity (or vigorous) exercise interventions (5, 18, 27), but the HR (91.1 ± 2.5 bpm) and ratings of perceived exertion (12.7 ± 1.1) values recorded during dynamic cycling sessions are defined as light to moderate intensity (22, 31). During dynamic cycling, the motor does the majority of the work to turn the pedals and less effort is required by the individual. The significant decrease in power in the dynamic group (31.2 ± 4.1), compared to the static group (8.0 ± 4.3), reflects this effort. The value of this rehabilitation paradigm is that it promotes significant improvement in PD symptoms with a reduced the risk of injury, excessive fatigue, and non-compliance. All of the individuals in the dynamic group were able to successfully complete three sessions without any unusual fatigue or injuries.

### Possible mechanisms

The improvement in Parkinson’s motor symptoms in the dynamic group was intriguing because individuals in the static group were working harder (higher HR and power) but showed no improvement in symptoms. These results suggest that motor improvement after dynamic cycling is not driven by purely cardiovascular or metabolic mechanisms (3). We propose that complex and variable sensory input during dynamic cycling increases sensory feedback from the periphery and subsequent activation of the basal ganglia circuits. Activation of these circuits could enhance central motor processing. Accurate voluntary movement requires somatosensory input from the periphery. Peripheral receptors, such as joint receptors, golgi tendon organs, muscle spindles, and cutaneous receptors, send information from the limbs to the cortex. Several studies have identified proprioceptive impairment in PD, specifically in muscle spindle responses, load sensitivity, and kinesthesia (12, 32–35). This suggests that deficits in peripheral afferent input or sensorimotor integration likely contribute to abnormal motor output in individuals with PD.

During dynamic cycling, proprioceptors measuring joint angles, muscle length and force, and cutaneous receptors on the bottom of the foot (36) would be activated. Improvements in motor function and mobility after bouts of cycling in individuals with PD could be due to increases in afferent input to the cortex. Several EEG studies in healthy individuals have shown that significant sensorimotor processing is present during active pedaling (37) and that high-cadence training promotes neural efficiency as defined with EEG spectral power analysis (38). This indicates that activation of proprioceptors with a high frequency but variable pattern may be important for symptom improvements in PD.

Bradykinesia, one of the most central cardinal symptoms of PD, may have significant origins in the alteration of scale perception as it relates to movement and may point to a possible underlying dysfunction in sensorimotor integration (12, 39). Our data with dynamic cycling suggest that the combination of (1) high-cadence cycling and (2) the introduction of variable cadence improve symptoms in PD, most notably rigidity and bradykinesia. The idea that dynamic cycling could invoke the retuning and integration of kinesthesia, as it relates to motor programing, is compelling. Naito has shown that kinesthetic input illusion activates primary motor cortex, as well as other related motor areas, including cingulate motor area and supplementary motor area (40), in healthy individuals. They also suggested that sensorimotor integration could occur directly in these motor regions. Thus, exploration of this mechanism by studying sensory changes in individuals with PD through the course of the adaptive dynamic cycling intervention has a high likelihood of yielding illuminating results regarding mechanisms of improved motor function.

Several studies have shown that bradykinesia and gait in PD can be improved with dynamic sensory cues (41–43). The theory of paradoxical kinesia, which suggests that motor action triggered by sensory stimuli circumvents damaged basal ganglia pathways (41, 44, 45), has been proposed as a mechanism for these improvements. In addition, research investigating the benefits of dancing in PD has suggested that the strong musical rhythms and asymmetrical movements in tango provide important sensory feedback cues that promote improvements in balance and gait (46–48). However, additional research examining the changes in proprioceptive sensitivity after dynamic cycling is necessary.

### Limitations

There are a few limitations to this study. We chose to exercise and test individuals in the “on” medication state in an effort to examine a true functional state. Individuals with PD would not exercise while “off” medication on their own. In addition, there is an increased risk of fall, injury, or discomfort during the “off” medication state. However, a recent exercise study by Prodoehl et al. (49) suggested that testing while “on” medication is adequate, as long as the timing of the last dose of medication relative to testing is controlled. In this study, we completed the pre-intervention and post-intervention testing at the same time of day and recorded when the last medication dose was taken in an attempt to minimize this variable. A second limitation of this study is a small sample size, which led to significant variability in responses within the groups. We did not want to limit our pool of participants by narrowing the inclusion criteria and, as a result, we had a wide range of disease severity and symptoms in our study. The pre-intervention UPDRS Motor III scores ranged from 11–40 in the static group (out of 108 possible) to 4–55 in the static group. Although participants were randomized into either dynamic or static cycling, the baseline UPDRS scores were different between the two groups. However, our statistics analyzed the baseline and post-intervention scores in each group independently to minimize the effect of this difference. Lastly, despite our hypothesized sensory-based mechanism of improvement, we did not measure sensory function directly in this study. Future studies will measure changes in proprioceptive sensitivity using a passive joint repositioning test (50).

## Conclusion

We believe that dynamic cycling provides variable sensory input to the basal ganglia that promotes improvements in motor speed and quality. The dynamic nature of this paradigm will allow for optimization of the therapy per individual through adaptive control mechanisms and over time. Due to the variation in responses to this therapy, additional work is needed to determine how dynamic cycling can be individualized for people with varying degrees and severity of symptoms. Future studies will test this theory by examining both motor and sensory function throughout the long-term dynamic cycling intervention.

## Author Contributions

AR was the primary designer of the study, oversaw data collection, data analysis, preparation of the manuscript, and agreed to be accountable for all aspects of the work. RP was primarily responsible for data collection and analysis and assisted in manuscript preparation. BW assisted in study design and data collection (UPDRS). FD and KL assisted in study design and were responsible for design and development of the dynamic cycle. All authors have given final approval of the version to be published.

## Conflict of Interest Statement

Provisional patent filed through KSU on 12/2014; full application has not yet been submitted; no royalties have been distributed.

## Funding

This work was funded by a National Institutes of Health Grant R21 HD068846 to Angela L. Ridgel.
